# Effectiveness of psychosocial school interventions in Finnish schools for refugee and immigrant children, “Refugees Well School” in Finland (RWS-FI): a protocol for a cluster randomized controlled trial

**DOI:** 10.1186/s13063-021-05715-6

**Published:** 2022-01-27

**Authors:** Reeta Kankaanpää, Sanni Aalto, Mervi Vänskä, Riina Lepistö, Raija-Leena Punamäki, Emma Soye, Charles Watters, Arnfinn Andersen, Per Kristian Hilden, Ilse Derluyn, An Verelst, Kirsi Peltonen

**Affiliations:** 1 Faculty of Social Sciences/Psychology, Tampere University, Tampere, Finland; 2 School of Education and Social Work, University of Sussex, FIN-33014 Tampere, Finland; 3 Section for Trauma, Catastrophes and Forced Migration - Adults and Elderly, Norwegian Centre for Violence and Traumatic Stress Studies, Oslo, Norway; 4 Department of Social Pedagogy, Ghent University, Ghent, Belgium; 5 Research Centre for Child Psychiatry, University of Turku, Turku, Finland; 6 INVEST Research Flagship Center, University of Turku, Turku, Finland

**Keywords:** Refugee, Immigrant, Adolescents, Multi-layered psychosocial interventions, Cognitive-behavioral treatment, Schools

## Abstract

**Background:**

Schools are natural environments in which to enhance young people’s social and emotional skills, mental health, and contact between diverse groups, including students from refugee and immigrant backgrounds. A layered or tiered provision of services is recommended as it can be effective to meet the needs of war-affected adolescents who variably show mental health problems (such as posttraumatic stress disorder (PTSD)). The current protocol describes the study design for a multi-layered intervention model. The study will test the effectiveness of two interventions: a teacher-training intervention In-Service Teacher Training (INSETT) combined with targeted cognitive-behavioral treatment-based Teaching Recovery Techniques (TRT) and a classroom-focused preventive intervention Peer Integration and Enhancement Resources (PIER). We analyze, first, whether the interventions are effective in decreasing psychological distress and increasing positive resources, i.e., prosocial behavior and resilience among refugee and immigrant students. Second, we analyze which student-, school-, and parent-related factors mediate the possible beneficial changes. Third, we look at which groups the interventions are most beneficial to.

**Methods:**

A three-arm cluster RCT with parallel assignment, with a 1:1:1 allocation ratio, is applied in 16 schools that agreed to participate in the Refugees Well School interventions and effectiveness study. Schools were randomized to three conditions of two active interventions and a waiting list control condition. Students, their parents, and teachers in intervention and control schools participated in the study at baseline before the interventions, after the interventions, and at 6 to 12 months after the interventions. The primary effectiveness criterion variables are psychological distress (SDQ) symptoms, resilience (CYRM-12), and prosocial behavior (SDQ).

**Discussion:**

The current study presents a recommended universal approach of layered interventions aiming to reduce psychological distress and increase resilience among refugee and immigrant students. A combination of promotive, preventive, and targeted interventions may offer a holistic, ecological intervention package for schools to better address the needs of the whole group.

**Trial registration:**

ISRCTN ISRCTN64245549. Retrospectively registered on 10 June 2020

## Administrative information

Note: the numbers in curly brackets in this protocol refer to SPIRIT checklist item numbers. The order of the items has been modified to group similar items (see http://www.equator-network.org/reporting-guidelines/spirit-2013-statement-defining-standard-protocol-items-for-clinical-trials/).

**Table:** 

Title {1}	Effectiveness of Psychosocial School Interventions in Finnish Schools for refugee and immigrant children, “Refugees Well School” in Finland (RWS-FI): a protocol for a cluster randomized controlled trial
Trial registration {2a and 2b}.	The study was registered with ISRCTN (ISRCTN64245549). Date assigned 10.6.2020, last edited 10.6.2020.
Protocol version {3}	Protocol version: 1 (2020-07-02)
Funding {4}	This project has received funding from the European Union’s Horizon 2020 research and innovation programme under grant agreement No 754849.
Author details {5a}	Faculty of Social Sciences/Psychology, Tampere University School of Education and Social Work, University of Sussex Section for Trauma, catastrophes and forced migration – adults and elderly, Norwegian Centre for Violence and Traumatic Stress Studies Department of Social Pedagogy, Ghent University
Name and contact information for the trial sponsor {5b}	Tampere University, Faculty of Social Sciences Kalevankatu 5, Linna Building, Unit of Psychology Tampere 33014 Finland +358 (0) 294 52 11, www.tuni.fi European Union Framework Programme for Research and Innovation, EU Framework Programme for Research and Innovation, Horizon 2020 - Research and Innovation Framework Programme
Role of sponsor {5c}	We have no conflicts of interest to disclosure.

Many refugee children and adolescents suffer from mental health problems such as high levels of PTSD, depressive, dissociative, and aggressive symptoms [1–3]. Yet, they can also be remarkably adaptative and resilient [4–6]. Augmenting research is available about effective ways to promote refugee and immigrant children’s positive adaptation in a new country and prevent risks of mental health and developmental problems [7–9].

Schools are natural environments to provide psychosocial support for minors [10–13]. Nevertheless, targeted school-based interventions have been criticized for their inability to cover the full range of multi-faceted problems refugee youth face in their everyday life [14–17]. Multi-layered or triad intervention models may match more accurately than single interventions with the needs of adolescents exposed to traumatic war experiences and refugee-related stress [18–20]. The present protocol describes the study design for a school-based multi-intervention model; combined interventions aim to promote resilience, prevent mental health problems, and reduce symptoms of an ongoing disorder especially among refugee and immigrant adolescents. The study will evaluate the effectiveness of these promotive, preventive, and focused interventions. Further, it will examine student-, school-, and home-related factors that may mediate or moderate the effect of interventions on student well-being.

## Effectiveness of multi-layered and targeted psychosocial interventions among refugee youth

Multi-layered intervention models typically involve three types of interventions: promotive universal psychosocial support interventions, preventive interventions provided to whole schools or classrooms, and targeted cognitive-behavioral therapies (CBT) [19, 21]. Although this approach is increasingly recommended, only three studies were detected to have evaluated the effectiveness of multi-layered interventions [22–24]. The results seem promising but are rather inconsistent and lack statistical power.

The first study [22] presented a practice-driven evaluation of a multi-layered community-based care package in four war-affected countries. Client satisfaction was high, and mental health problems were reduced moderately. Yet, service providers reported significant levels of distress related to service delivery. The second study [23] assessed the effectiveness of a school-based mental health program for traumatized immigrant children and adolescents in the USA. All students were provided with an array of clinical services including CBT, supportive therapy, and coordinating services. A trauma-focused (TF) CBT was implemented with a subset of students. The greater quantity of CBT and supportive therapy was associated with increased psychosocial functioning, and a greater quantity of coordinating services was associated with decreased PTSD symptoms. TF-CBT was associated with both improved functioning and decreased PTSD symptoms. The results thus suggest that the comprehensive school-based model was effective and that different service components resulted in specific outcomes. The third study [24] examined the effectiveness of a four-tiered program for young Somali refugees in the USA. The intervention elements included community resilience building, school-based universal early intervention, and targeted treatment for students suffering from severe psychological distress. The authors reported that students across all tiers of the program demonstrated improvements in mental health and resources. Yet, the sample size per tier was small, and comparative analyses between tiers were not possible to conduct.

School- or classroom-based promotive and preventive interventions are often delivered through teachers’ education. Developmental theories for ecological systems suggest enhancing teachers’ cultural competence and increasing their awareness of the impact of trauma on learning [25]. Teachers have a pivotal role in helping refugee and migrant students to improve their mental health [26–30].

Focused psychosocial school interventions have shown a beneficial impact on trauma-affected refugee and immigrant children. This has been indicated by decreased PTSD, depressive, and anxiety symptoms and functional and peer problems [20, 31–33]. However, effect sizes vary between moderate and very high across studies, and also null results have emerged [34–36]. Cognitive-behavioral treatments with trauma-focus (TF-CBT) belong to the group of focused psychosocial interventions. They can involve several therapeutic elements such as learning to process past traumatic events verbally or multi-modally, expressing painful emotions, and constructing a new narrative from children’s shattered experiences in a new and safe environment. Other common intervention elements are the use of various creative techniques including drama, music and drawing, shared activities within the classroom, and interaction with peers [37].

School-based interventions for refugee and immigrant children and adolescents can attune anxiety and depression and reduce PTSD symptoms [32, 38]. Interventions using CBT-based methods have been shown especially effective with trauma-affected children [39]. Interventions with creative elements of writing, drawing, or drama are found to decrease immigrant children’s and adolescents’ mental health problems [38] and to increase positive resources such as effective coping and hope [40].

In the current study, the promoting intervention is In-Service Teachers’ Training (INSETT) which aims at enhancing teachers’ cultural competence and self-efficacy through training. Teaching Recovery Techniques (TRT) is the targeted, TF-CBT-based intervention. A classroom-based, preventive intervention called Peer Integration and Enhancement Resources (PIER) provides tools to increase and reinforce multi-cultural friendships and to create a safe and welcoming classroom environment for adolescents from migrant and refugee backgrounds.

## Mediating and moderating effects of psychosocial interventions

Four mechanisms of change were found strong empirical evidence in the context of psychosocial interventions aiming to improve well-being, mental health, and resilience among children affected by war [41]. The first mechanism was *building family and caregiver capacity*, which involved support to families, caregivers, and practitioners to enable them to enhance child well-being through psychoeducation, dialog, and self-care. The second mechanism was *strengthening family and caregiver relationships with children facing traumatic stress*. Strengthening relationships was especially important in collectivist cultures where family relationships form a core resource. The third mechanism concerned children’s capacities: *improving active problem solving, effective coping, and mastery of traumatic experiences*. The fourth mechanism was *therapeutic rapport in targeted interventions*, and it was indicated by trusting therapeutic relationships and a safe environment for disclosure of traumatic experiences. It is noteworthy that only moderate evidence was found for TF-CBT-based mechanisms, such as processing trauma through reconstructing harmful cognitions, framing narratives, playing, or learning effective regulation of painful emotions. Also, interventions focusing on strengthening community and cultural resources, values, and rituals did not gain high-quality support among war-affected children.

A study based on the ecological resilience framework found that increased social support mediated the intervention impact resulting in reduced PTSD symptoms. Change in hope or coping strategies did not mediate treatment effects on PTSD symptoms [42]. Using metadata, one study [43] found only psychosocial functioning (in personality, social, and academic domains) to mediate intervention impact on mental health among children in low-resource humanitarian settings. Instead, the hypothesized effective coping strategies, social support, or positive attitudes of hope were not significant mediators of intervention impacts.

## Objectives

The first aim of this research project is to test whether two school-based psychosocial intervention arms, (a) the combined In-Service Teacher Training (INSETT) and Teaching Recovery Techniques (TRT) and/or (b) the Peer Integration and Enhancement Resource (PIER), are effective in improving the mental health among refugee, migrant, and Finnish native adolescents. The criteria for intervention effectiveness are reducing psychological distress (internalizing and externalizing symptoms) and increasing prosocial behavior and resilience compared to adolescents in control schools. The second aim is to examine which adolescent- and/or school-related factors could explain the effectiveness of these two arms of school-based psychosocial interventions (mediation analysis). Third, we analyze whether the adolescent- and family-related preconditions differ in the effectiveness of these two intervention arms (moderation analysis).

The research questions and hypotheses are as follows:

1. Do psychosocial school interventions have a positive effect on the mental health of immigrant adolescents? In more detail, the aim is to compare the impact of INSETT+TRT and PIER to the waiting list control group on internalizing and externalizing symptoms and prosocial behavior and resilience.
  - We hypothesize that internalizing and externalizing symptoms will decrease statistically significantly only among adolescents participating in the INSETT+TRT and PIER interventions and not in the control group from baseline (T1) to 6-month (T2) and 12-month (T3) follow-ups.
  - We hypothesize that prosocial behavior and resilience will increase only among adolescents participating in the two arms of interventions and not in the control group.
2. Do different adolescent- and school-related factors explain (mediate) the effects of the INSETT and PIER interventions on adolescent mental health?
  - Concerning INSETT, we hypothesize that participation of teachers increases their multi-cultural awareness, sense of self-efficacy, and work engagement and decreases their work stress, which in turn are associated with the decreased internalizing and externalizing symptoms and increased prosocial behavior and resilience among the adolescents.
  - We hypothesize that adolescents’ participation in PIER intervention is associated with their increased social support, feeling of belongingness, number of inter-ethnic friendships, and satisfaction on friendships, which in turn is associated with decreased internalizing and externalizing symptoms and increased prosocial behavior and resilience.
  - We hypothesize that adolescents’ participation in TRT intervention is associated with decreased PTSD symptoms of intrusion and avoidance, which in turn is associated with decreased internalizing and externalizing symptoms and increased prosocial behavior and resilience.
3. How do parent- and adolescent-related factors moderate the effectiveness of the two arms of school-based psychosocial interventions of INSET+TRT and PIER?
  - We hypothesize that good parental mental health and parents’ high sense of competence and confidence in parenting are associated with a statistically significant positive intervention-induced change in adolescents’ mental health (i.e., decreased internalizing and externalizing symptoms and increased prosocial behavior and resilience).
  - We hypothesize that adolescents who report the low frequency of daily stressors and low perceived discrimination show more statistically significant positive intervention-induced change in their mental health (i.e., decreased internalizing and externalizing symptoms and increased prosocial behavior and resilience).

## Trial design

The study is a three-arm clustered parallel assignment, quasi-randomized, controlled trial (RCT) comparing INSETT+TRT and PIER to the wait list control group. The cluster is designed by the school. The schools were allocated into intervention groups or a control group using quasi-randomized design: The voluntary schools were first grouped based on whether they offered an introductory class or not. The TRT is targeted at recently arrived youth with a refugee background, and the PIER is targeted at mixed classes. The schools in the two groups were then randomly allocated to either intervention or control conditions using a random number generator [44]. The allocation aimed at equal numbers of schools in each condition. Due to the low participation rate, recruitment of schools was continued, which resulted in three additional schools all with introductory classes. The three additional schools were thus randomized either into the INSETT+TRT or control group. Blinding of participants, intervention providers, outcome assessors, or data analysts was not possible due to the explicit nature of interventions and a low number of project members. The decisions of data collection, recruitment, and training are discussed with other partners in the RefugeesWellSchool consortium but allowing each country to adopt the processes in the national framework. The control schools will receive the INSETT intervention toolkit after the research part of the project has ended. The trial aims to follow SPIRIT guidelines in reporting. Table 1 presents the SPIRIT figure, and the checklist can be found as an attachment.

**Table 1:**
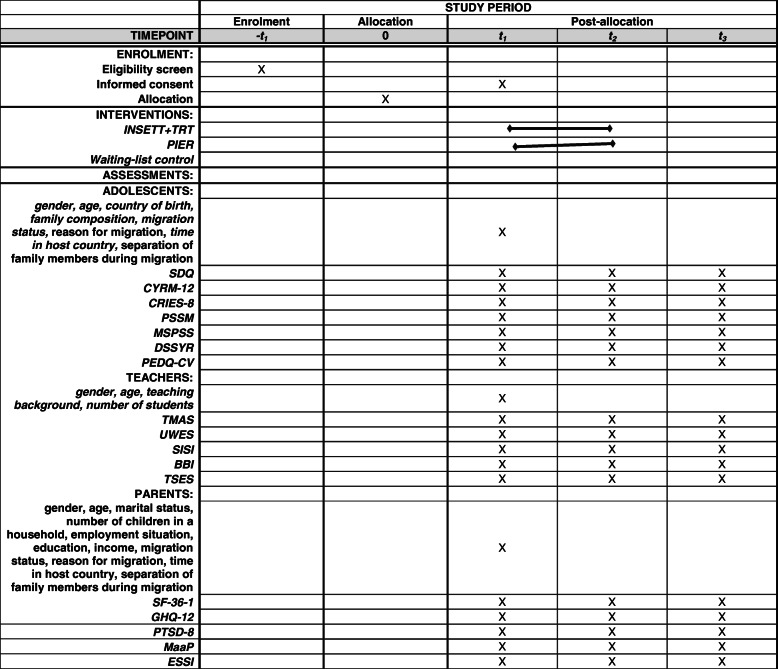
Study period for implementing interventions, collecting data (T1, T2, T3), and specific measures for adolescents, teachers, and parents

## Study setting and participants

Power calculations were conducted before the data collection started. With alpha set at 0.05, power at 0.80, and rho = 0.05 (the intra-cluster correlation), a sample size of at least 40 clusters (i.e., schools) with 25 youths each was identified as necessary. Therefore, 500 youths per study arm (i.e., intervention or wait list control) were needed, resulting in 1500 total participants. To account for an expected drop-out rate of 20%, 1800 youths would have been recruited in total. To facilitate the recruitment of so many participants, recruitment was split between the Finnish study team and our team partners in Sweden.

**Fig. 1:**
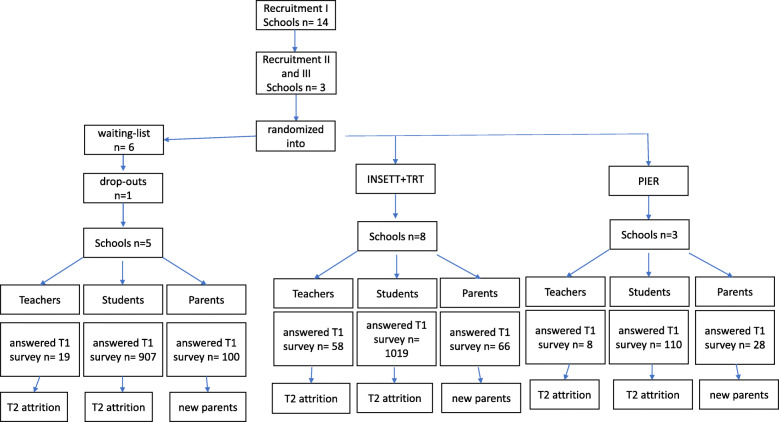
The flowchart of experimental vs. control situations in the study design

The interventions were implemented in lower secondary schools with grades 7–9 and/or introduction classes for newcomer youth. We focused on immigrant youth who have resided in Finland for less than 6 years. Figure 1 presents the flowchart of the recruitment. Seventeen schools expressed their interest in participating in the study, but after allocation, one school dropped out. According to the randomization, eight schools were allocated into the INSETT+TRT intervention, three schools were allocated into PIER, and five schools served as waiting list controls. Also, Fig. 1 reports the numbers of students, teachers, and parents who answered the survey in the first round of data collection.

## Recruitment and sampling

The recruitment of schools was based on three aspects. First, voluntariness, i.e., their (headmasters and teachers’) self-defined need for intervention, knowledge, and training on psychosocial skills while teaching refugee and immigrant adolescent students. For that purpose, the first recruitment was through advertisement in the national teachers’ magazine (Opettaja) and email lists to school administration in every municipality in Finland. Second, the recruited and voluntary schools should have a considerable share of refugee and immigrant background pupils in secondary school classes estimated as 30–50% in each class. Third, the schools accepted the possibility of serving as a waiting list school until the next school year when they would be provided the INSETT intervention in electronic form. The first wave of recruitment was complemented with about 100 phone calls to headmasters of schools residing in the metropolitan or otherwise immigrant-dense areas of larger cities in Finland. In the second wave of recruitment, phone calls and e-mail communications were established to invite schools close to asylum centers in numerous towns and municipalities in Finland. However, most extra work to reach schools near asylum centers and the metropolitan area was not successful as schools informed that they already had several general interventions (such as anti-bullying whole-school programs) or trauma and refugee-focused programs (such as cultural coaching programs). As a result of the two waves of recruitment, 16 schools agreed to participate in the Refugees Well School study.

Table 1 presents the study period for implementing interventions, collecting data (T1, T2, T3), and specific measures for adolescents, teachers, and parents. Baseline data was collected before the interventions, INSETT+TRT and PIER interventions were implemented between September 2019 and January 2020, post-intervention data collection took place around February 2020, and the follow-up data will be collected in August 2020. INSETT will be provided to waiting list control schools in October 2020.

## Eligibility criteria

The unit of recruitment was schools. As both the INSETT+TRT and PIER interventions aim at enhancing school belonging, high peer quality, interethnic friendships, and understanding and protecting cultural diversity, we included both native-Finnish and refugee-immigrant adolescents in the interventions and effectiveness study. The only exclusion criterion for schools was not to have other similar psychosocial interventions running at the same time. For students, there were no exclusion criteria, and all who signed the informed consent could participate. The 16 schools were located across the country of Finland and represented mainly refugee and immigrant-dense urban areas. One school is in the south, (the metropolitan area), two in the east, three in the middle, two in the west, and three in northern Finland. If a participant should not want to continue participating in the study, she/he may resign simply by informing the researchers, and the given participant data would be deleted from the system. If intervention participation would cause harm to a participant, she/he would be advised to appropriate services. The participation was not expected to cause harm to the participating individuals, since the interventions had been shown to increase well-being and decrease mental health symptoms in the previous studies [45]. In the baseline data collection phase, the researchers stayed at the study site and were ready to discuss with the students if they felt distressed after filling the survey. Reported experiences of caused harm will be stored anonymously and described in the results.

## The interventions

In-service Teacher Training (INSETT) is developed by Lutine de Wal Pastoor and it is designed for lower and upper secondary school teachers and counselors in introductory-, preparatory-, and ordinary classes [46]. The INSETT aims at enhancing teachers’ cultural competence concerning refugee experience and self-efficacy via teachers’ education, cultural awareness, and peer support practices. After INSETT, we expect teachers’ improved skills to induce positive changes in three key areas: [1] promoting and supporting mental health and well-being among trauma-affected students with refugee and migration backgrounds, [2] encouraging students in positive interethnic relationships in linguistically and culturally diverse school settings, and [3] fostering relationships between teachers and parents/caregivers to promote co-operation and involvement with schoolwork. The teachers’ training and awareness building is expected to benefit the well-being and sense of belonging of refugee and immigrant students directly and their caregivers indirectly.

The intervention involves a combination of two seminar days with lectures, discussions, exercises, and exchange of ideas and experiences between participating teachers, and an online training course to be completed by teachers individually. The INSETT intervention provides seminar topics, materials, and literature to prepare thematic sessions, and slides [46], and the online training course *Providing support to refugee minors* by the Augeo Foundation in the Netherlands [47] and extensive local-language material to schools in Finland, Norway, and Sweden.

*Peer Integration and Enhancement Resource (PIER)* is delivered by lower and upper secondary school teachers, counselors, and special teachers in school classes including both native and refugee and immigrant students. The PIER aims at supporting safe and positive peer interactions and social relationships in multi-ethnic schools. This is expected to happen through group exercises that focus on strengthening the sense of belonging, empathy, and role-taking; learning from each other; and getting and giving more social support. The manualized intervention consists of eight sessions ranging between 45 and 90 min. Sessions include structured welcoming and ending rituals and multi-modal group activities such as cartoon drawing, role-play, movies, and drama. Sessions also include practices of reflecting various identities, migration, and racism.

The school staff delivering the PIER participated in a 2-day training. The first day included practicing each intervention session and going through the resource material. The second day included sharing experiences and valuable ideas to improve the intervention.

*Teaching Recovery Techniques (TRT)* is a TF-CBT-based, manualized group treatment developed by the Children and War Foundation [48]. The core aim of this intervention is to create a sense of safety and increase feelings of competence and shared hope. In more detail, the aims are to reduce PTSD symptoms of intrusion, avoidance, and hypervigilance; increase resilience; stabilize trauma reactions; and provide practical techniques and strategies to deal with traumatic memories, physical and behavioral arousal, and withdrawal. Treatment elements include psychoeducation (in playful and multi-modal ways), normalizing reactions to trauma, working with nightmares, mastering intrusive memories and trauma reminders with framing techniques, and dealing with avoidance and arousal with scaling techniques. Homework such as sleep hygiene or reflective observation of trauma reminders is an essential part of the TRT.

The TRT consists of five sessions of 90 to 120 min including skills training, rehearsal, and homework, and the handbook provides each session with several tools, techniques, and procedures. The two sessions for parents or caregivers include information about various responses to traumatic events and effective coping strategies. School personnel delivering the TRT participate in 2-day intensive training provided by a licensed trainer either from the Children and War Foundation or from Finland. Qualified trauma psychologists offer work counseling to TRT group leaders during the intervention.

## Data collection and management

Survey data is collected online from students, teachers, and parents, and informed consent is collected both on paper and online at the beginning of the survey. Foreign-speaking parents receive both online and paper surveys, and the informed consent form is both on paper and online. The online survey is managed using a secure online survey tool [49], and all identifiable information will be stored separately from the research data. The researcher team will collect informed consent first in schools and later via mail. To avoid attrition, participants will receive a personal invitation via e-mail and two reminders to answer the post-intervention (T2) and follow-up (T3) surveys. The RWS consortium will manage and store the research data and will have a common data management plan available online.

## Measures

Table 2 presents the measurements taken at each three assessment points, i.e., before, during, and after the interventions. Model form of the informed consent and detailed description of questionnaires is available from the first author upon request. All outcome measures will be measured as the difference in the change in score between the two groups, i.e., intervention and control conditions. The primary time point of interest is from baseline (T1) to post-intervention (T2), and the secondary time point is from baseline (T1) to follow-up (T3). Measures will be aggregated as means.

**Table 2:** Study constructs, measures, and questionnaires of students, parents, and teachers

	Construct	Measure	Assessment tool
Students	Youth mental health	PTSD symptoms	Children’s Revised Impact of Events Scale (CRIES-8)
		Internalizing and externalizing problems and prosocial behavior	Strengths and Difficulties Questionnaire (SDQ)
		Positive development and resilience	Child and Youth Resilience Measure (CYRM-12)
	Associated factors of youth mental well-being	Experience of the number of stressors in daily life	Daily Stressors Questionnaire (DSSYR; Vervliet, Derluyn, and Broekaert, unpublished)
		Social support	Multidimensional Scale of Perceived Social Support (MSPSS)
		Existence of interethnic friendships and satisfaction with friendships	Items developed for this study
		Discrimination	The Perceived Ethnic Discrimination Questionnaire Community Version (PEDQ-CV)
		School belonging	The psychological sense of school membership among adolescents (PSSM)
	Sociodemographic factors	Gender, age, country of birth, migration status, time in the host country, and family composition	
Parents	Parental health	Self-reported health	One item of the SF-36
		Mental health	General Health Questionnaire (GHQ-12)
		PTSD symptoms	PTSD-8 Questionnaire
	Parent experiences of inclusion	Experience of discrimination	Brief Perceived Ethnic Discrimination Questionnaire – Community version (PEDQ-CV)
		Social support	Enriched Social Support Instrument (ESSI)
	Family relations	Parenting	Me as a Parent (MaaP)
	Sociodemographic factors	Sex, age, marital status, number of children in a household, employment situation, education, income, time in the host country, migration status, the reason for migration, and separation of family members during migration	
Teachers	Cultural competence	Multi-cultural awareness and understanding	Teacher Multicultural Attitude Scale (TMAS)
	Self-efficacy	Teachers’ self-efficacy	Teachers’ Sense of Efficacy Scale (TSES)
	Stress and work engagement	Stress symptoms	Single Item Stress Index (SISI)
		Work exhaustion/burnout	Bergen Burnout Inventory (BBI)
		Work engagement (vigor, dedication, absorption)	Utrecht Work Engagement Scale (UWES)
	Sociodemographic factors	Sex, age, teaching background, and number of students	

### Primary measures

*Psychological distress*, i.e., *internalizing and externalizing problems*, is measured with the self-report version of the Strengths and Difficulties Questionnaire (SDQ) [50] for 11–17-year-olds. SDQ is a screening questionnaire that measures 25 attributes divided into either two sub-scales of internalizing and externalizing problems or five subscales: emotional symptoms, conduct problems, hyperactivity-inattention, peer problems, and prosocial behavior. All questions are asked on a scale of “not true,” “somewhat true,” or “certainly true.” “Somewhat true” is always scored as 1, but the scoring of “not true” and “certainly true” varies with the item. For each of the five scales, the score can range from 0 to 10 if all items are completed. Goodman [51] reports SDQ to have acceptable validity and reliability (lowest in peer problems *α* .41 and highest in total difficulties *α* .80). In addition, research shows good validity for internalizing scales indicated by correspondence between children, parents, and teacher reports and clinical review [52].

*Resilience* is measured with an adapted, focus group-based Child and Youth Resilience Measure (CYRM-12) [53]. CYRM was originally designed as a 28-item measure for youth aged 9 to 23 years old. A shortened resilience measure (CYRM-12) consists of 12 items, which are scaled as 5 points (1 “not at all” to 5 “a lot”). A total score is created by summing up the score of each item. CYRM is a questionnaire exploring the individual, relational, communal, and cultural resources that may bolster the adolescents’ resilience. The participant reports on a 5-point scale as to what extent he/she feels he/she has certain resources. Preliminary results show tentative content validity of the CYRM-12 to merit its use as a screener for resilience processes in the lives of adolescents, and reliability was *α* .84.

*Posttraumatic stress symptoms (PTSD)* are screened with the Children’s Impact of Events Scale (CRIES-8) [54–56]. CRIES-8 has two sub-scales, namely intrusion and avoidance, and it is designed to be used in children aged 8 and above. The eight items are scored on a 4-point scale (0 “not at all,” 1 “rarely,” 3 “sometimes,” 5 “often”). The total score is the sum of scores from the two sub-scales. The screening cutoff is on ≤ 17 points. CRIES-8 has been applied, and its factors proved as robust in a variety of cultures. It has good construct validity and quite a stable factor structure, it correlates well with other indices of distress, and it has been used to screen very large samples of at-risk children following a wide range of traumatic events [57]. Another study [58] reported CRIES-8 to have good internal consistency (*α* .86).

### Other measures

*School belonging* is measured with the Psychological Sense of School Membership (PSSM) among adolescents [59]. *Social support* is measured with a Multidimensional Scale of Perceived Social Support (MSPSS) [60]. *Interethnic friendships* are measured as the number of interethnic friendships and satisfaction with friendships; measures were developed for this study. *Daily stressors* are measured with the Daily Stressors Questionnaire (DSSYR; Vervliet, Derluyn, and Broekaert, unpublished). *Discrimination* is measured with the Perceived Ethnic Discrimination Questionnaire Community Version (PEDQ-CV) [61].

*Teachers’ multicultural skills* are measured with the Teacher Multicultural Attitude Scale (TMAS) [62]. *Teachers’ self-efficacy* is measured with the Teachers’ Sense of Efficacy Scale (TSES) [63]. *Teachers’ work engagement and stress* are measured with three measures: Utrecht Work Engagement Scale (UWES) [64], Single-Item Stress Index (SISI) [65], and Bergen Burnout Inventory (BBI) [66].

*Parents’ general and mental health* is measured with one item of the SF-36 [67], General health Questionnaire (GHQ-12) [68], and PTSD-8 Questionnaire [69]. *Parenting* is measured with the Me as a Parent (MaaP) [70], *parents’ social support* is measured with the Enriched Social Support Instrument (ESSI) [71], and *parents’ perceived discrimination* is measured with the same as with adolescents [61].

### Background variables

Adolescents report their gender, age, country of birth, and family composition, and migrant students also report migration status, the reason for migration, time in the host country, and separation of family members during migration. Teachers report their gender, age, teaching background, and the number of students they teach. Parents report their gender, age, marital status, number of children in a household, employment situation, education, and subjective income, and immigrant parents also report migration status, the reason for migration, time in the host country, and separation of family members during migration.

The categories for natives, immigrants, and refugees will be formed using the question reason for migration. Natives will be categorized as those who are born in Finland. Immigrants will be categorized as those who respond they had come to Finland because of their parents’ temporary or permanent work. Refugees will be categorized as those who report they were fleeing war, persecution, or danger.

## Analytic strategy

Due to the sampling procedure, our data will be clustered in two levels: pupils are nested in 134 classes, and classes in 16 schools. The data contains variables from individual, class, and school levels, and the interventions are given at the school level. All the indicators of outcome variables are measured at all three time points at the individual level. T1 was collected before the intervention, T2 20 weeks after the intervention started, and T3 as a follow-up 52 weeks after the beginning of the intervention. We expect the intervention to change the levels in outcomes from T1 to T2, and the new levels to remain the same through T3 follow-up.

**Fig. 2:**
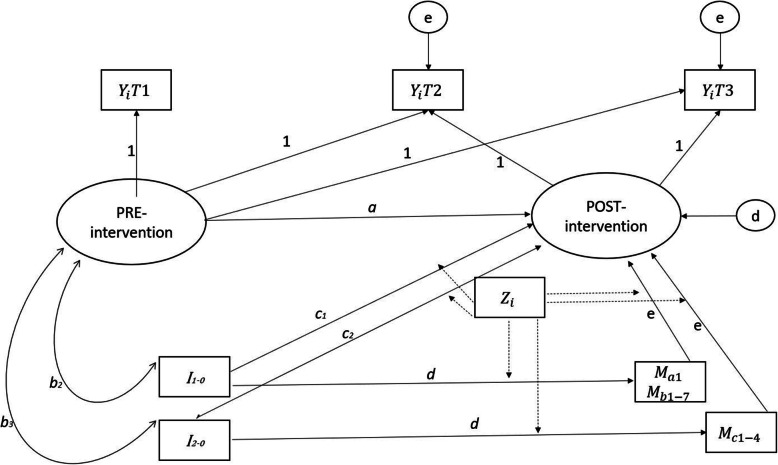
Latent growth curve theoretical model for analysis

The structure of the data requires the use of structural equation (SEM) methods combining longitudinal, multi-level, path analytic models with confirmatory factor analysis. The psychometric properties of the outcome variables, including measurement invariance between time points T1, T2, and T3, are tested before constructing summary scales using the most reliable items. The scales are then specified as manifest variables in the models. Alternatively, latent constructs are used, depending on the complexity of the final model specifications. The core model in all our analyses will be a version of latent growth curve model [72, 73], that is also known as intervention model [74] due to the coding of the fixed parameters in the model, which are responsible for the shape of the constant growth trajectories at the individual level (the 1’s in Fig. 2). The latent growth parameters will be estimated with the Mplus 8.0 software using a robust maximum likelihood estimator (MLR), which allows non-normal continuous indicators to be reliably analyzed. Additionally, MLR is a full-information estimator allowing the use of missing data without any separate imputations. The validity of the MAR assumption is scrutinized, and if needed, auxiliary variables predicting missingness will be added into the models. Demographic and baseline characteristics will be summarized using means and standard deviations for quantitative variables and percentages for categorical variables.

Explanations: Primary outcomes—*Y*_1_ = SDQ (difficulties), *Y*_2_ = SDQ (strengths), and *Y*_3_ = CYRM; intervention status—*I*_0_ = waiting list control group, *I*_1_ = INSETT+TRT, and *I*_2_ = PIER; possible moderators—*Z*_1_ = SF-36, *Z*_2_ = GHQ-12, *Z*_3_ = PTSD-8, *Z*_4_ = MaaP; *Z*_5_ = PEDQ-CV (parent), *Z*_6_ = PEDQ-CV (adolescent), *Z*_7_ = DSSYR, *Z*_8_ = (daily stressors), and *Z*_8…*n*_ = sociodemographics. Expected mediators: INSETT Teachers (competence)—Mb_1_ = TMAS, Mb_2_ = TSES, Mb_3_ = SISI, Mb_4_ = BBI, Mb_5_ = UWES, Mb_6_ = Trust, and Mb_7_ = classroom atmosphere; TRT Adolescents (trauma)—Ma_1_ = CRIES-8; PIER Adolescents (social)—Mc_1_ = MSPSS, Mc_2_ = PSSM, Mc_3_ = PEDQ, and Mc_4_ = interethnic friendships.

In the core model (Fig. 2), there are two latent state variables, measuring true scores of PRE- and POST-intervention levels of the outcomes. The PRE-intervention state is indicated by the measured T1 variable without time-related measurement error because there is only one measurement point. In the case of POST-intervention state, there are two indicators, enabling the modeling of measurement error. The loadings of the indicators in the measurement part of the model are fixed to 1’s, meaning that the trajectory from T1 to T3 has the same shape for every individual. Only the level of PRE-intervention state and the difference between PRE- and POST-intervention states are random so that the means and variances of these variables within and between the clusters can be estimated. As predictors of the POST-intervention state, our model specifies the PRE-intervention state (path coefficient *a*, which is anticipated to be negative due to floor and ceiling effects), a dummy for intervention/control group membership (direct effect *c*), and potential mediating variables (path coefficient *e*, implying indirect effect d*e for intervention-control-group difference). The covariance between the PRE-intervention state and the dummy for intervention/control group membership (parameter *b*) is supposed to be 0, indicating that the randomization was successful. Moderation mechanisms are investigated by estimating the model simultaneously in groups created according to categorized moderator variables (multi-group SEM). Finally, the simple estimates of total intervention effects are calculated from a model without mediators and moderators (parameter *c* divided by the variance of POST-intervention latent variable, giving an equivalent to Cohen’s *d*).

## Discussion

Most psychosocial interventions are targeted at immigrant children generally or to those who suffer from PTSD or other symptoms. There is a lack of universal interventions embedded in classrooms with both immigrant and native peers together [9, 19, 38]. Professionals recommend combining both preventive universal psychosocial elements and targeted clinical therapies, tailored according to the needs of immigrant children [18, 20, 32]. The number of immigrant children with an increased risk for mental health problems is growing. To understand the extended effect of school-based interventions, we need to broaden the research to include also information about child-, school-, and family-related factors [32, 38]. Similarly, the knowledge of what works for whom, why, and where would be of great importance when tailoring effective treatment for immigrant children. In trauma research, the relevant questions are about the factors and processes (or mechanisms) that explain (statistically mediate) the impact of war trauma on children’s and adolescents’ mental health and who are most vulnerable to negative trauma impacts, and why [75]. Analogously, in intervention studies, it is critical to know what explains the potential change and to whom interventions work and why.

As a limitation to the study, unfortunately, the recruitment did not reach the needed number of clusters for adequate power of 80%. The problems in the recruitment may have been because many schools had other ongoing interventions or had just participated in similar training. Some schools with a long history of teaching immigrant students doubted the intervention could provide them with any new tools. In more rural schools near asylum centers, the number of immigrant students had decreased rapidly after the great influx in 2015, and the intervention was thus not seen as necessary anymore. The low power will limit our chances of finding statistically significant effects on a school level.

The COVID-19 outbreak did not affect the training of the facilitators for the INSETT, TRT, or PIER interventions in Finland, as they were conducted in September 2019 to January 2020. However, the implementation of the TRT failed in the five schools that had planned the group sessions in spring 2020. The baseline assessment was conducted in classrooms as planned (August to September 2019), but due to the COVID-19 school closure, the timing of the 6- to 12-month assessments was delayed. Students were individually contacted by text messages, and they responded online, which was not as effective as conducting the data collection in classrooms.

## Dissemination

The results of this trial will be submitted for publication during 2021–2023, and publications will be made open access. The authorship of future trial publications will be determined by the research group, and other members of the H2020 consortium will be invited accordingly. Professional language editing will be used. The INSETT and PIER Finnish-language intervention manuals will be prepared open-access to schools. The data sets generated during the study will not be made open access but can be requested from the first author and according to the ethical approval.

Horizon 2020 did not influence the research design, conduct, or analysis. Also, the academic publications in national and international journals will not be influenced by the funder. However, the reports and deliverables requested by the EU will be offered as requested.

## Trial status

This is the first version of the study protocol (dated 1 July 2020). Recruitment began in January 2019 and was completed in August 2019. The data collection ended in September 2020, and the protocol was submitted before that.

## Data Availability

RWS consortium will manage and store the research data and will have a common data management plan available online.

## Abbreviations

BBI: Bergen Burnout Inventory
CBT: Cognitive-behavioral therapy
GHQ-12: General Health Questionnaire
CRIES-8: Children’s Impact of Events Scale
CYRM-12: Child and Youth Resilience Measure
DSSYR: Daily Stressors Questionnaire
ESSI: Enriched Social Support Instrument
INSETT: In-Service Teacher Training
MaaP: Me as a Parent
MAR: Missing at random
MLR: Robust maximum likelihood
MSPSS: Multidimensional Scale of Perceived Social Support
PEDQ-CV: Perceived Ethnic Discrimination Questionnaire Community Version
PIER: Peer Integration and Enhancement Resources
PSSM: Psychological sense of school membership among adolescents
PTSD: Posttraumatic stress disorder
RCT: Randomized control trial
SDQ: Strengths and Difficulties Questionnaire
SEM: Structural equation modeling
SISI: Single-Item Stress Index
TF-CBT: Cognitive-behavioral treatment with trauma-focus
TSES: Teachers’ Sense of Efficacy Scale
TMAS: Teacher Multicultural Attitude Scale
TRT: Teaching Recovery Techniques
UWES: Utrecht Work Engagement Scale
